# Phase 2 dose-expansion trial of OBI-3424, a DNA-alkylating prodrug, in patients with advanced solid tumors expressing AKR1C3

**DOI:** 10.1093/oncolo/oyag254

**Published:** 2026-07-02

**Authors:** Apostolia Maria Tsimberidou, Claire Verschraegen, Darren Sigal, Heinz-Josef Lenz, Howard Hochster, Mehmet A Baysal, Abhijit Chakraborty, Ingly Lee, Koenuel Ristoski, Dong Xu

**Affiliations:** Department of Investigational Cancer Therapeutics, The University of Texas MD Anderson Cancer Center, Houston, TX, United States; Division of Medical Oncology, The Ohio State University Comprehensive Cancer Center, Columbus, OH, United States; Scripps Clinic and Scripps Cancer Center, La Jolla, CA, United States; Division of Medical Oncology, Norris Comprehensive Cancer Center, Keck School of Medicine, University of Southern California, Los Angeles, CA, United States; Rutgers Cancer Institute, New Brunswick, NJ, United States; Department of Investigational Cancer Therapeutics, The University of Texas MD Anderson Cancer Center, Houston, TX, United States; Department of Investigational Cancer Therapeutics, The University of Texas MD Anderson Cancer Center, Houston, TX, United States; OBI Pharma USA, Inc., San Diego, CA, United States; OBI Pharma USA, Inc., San Diego, CA, United States; OBI Pharma USA, Inc., San Diego, CA, United States

**Keywords:** OBI-3424, pancreatic cancer, solid tumors, phase 2 clinical trial, safety, efficacy

## Abstract

**Background:**

OBI-3424 is an investigational small-molecule prodrug. In a dose-escalation trial, the OBI-3424 recommended phase 2 dose (RP2D) was 12 mg/m^2^ administered every 21 days. In this phase 2 dose-expansion trial, we evaluated the safety and efficacy of OBI-3424 in pancreatic adenocarcinoma and other solid tumor types (“basket” cohort).

**Methods:**

Patients with advanced solid tumors and an AKR1C3 IHC H-score of ≥ 100 were treated at the RP2D of OBI-3424. Tumor response, progression-free survival (PFS), overall survival (OS), and treatment-emergent adverse events (TEAEs) were assessed (www.clinicaltrials.gov NCT03592264).

**Results:**

Of the 29 patients treated, 26 were evaluable for response (pancreatic adenocarcinoma, *n* = 10; basket cohort, *n* = 16). In the pancreatic adenocarcinoma cohort, stable disease (SD) was observed in 40.0% of patients, and the median PFS and OS durations were 1.35 months and 3.8 months, respectively. In the basket cohort, the objective response rate was 6.3% (1 of 16 patients had a partial response), 50% of patients had SD, and the median PFS and OS durations were 2.53 months and 5.32 months, respectively. The most common TEAEs in both cohorts were anemia, fatigue, and thrombocytopenia.

**Conclusion:**

While OBI-3424 demonstrated a favorable safety profile, limited efficacy led to early trial termination.

Lessons learned

- In this first phase 2 trial, OBI-3424—a small-molecule prodrug selectively activated by AKR1C3 in the presence of NADPH to become a potent DNA-alkylating agent—was evaluated in patients with advanced solid tumors, demonstrated a favorable safety profile. The most common treatment-emergent adverse events were cytopenia and fatigue.
- Of the 29 patients treated, 26 were evaluable for response (pancreatic adenocarcinoma, *n* = 10; basket cohort, *n* = 16). The disease control rate was 40.0% in the pancreatic adenocarcinoma cohort and 56.3% in the basket cohort. The best overall response was a PR, observed in 1 patient (3.8%) with urachal adenocarcinoma.
- The trial was terminated early due to a lack of clinical efficacy, likely attributed to the moderate-to-high AKR1C3 H-score that was required for enrollment, inability of OBI-3424 to efficiently reach AKR1C3-rich targets to induce tumor cell death, and the complexity of tumor microenvironment particularly in pancreatic cancer, which is resistant to therapy.

## Drug information

Aldo-keto reductase family 1 member C3 (AKR1C3) is a nicotinamide adenine dinucleotide phosphate (NADPH)-linked oxidoreductase that reduces aldehydes and ketones to their corresponding alcohols.^1^^,^^2^ While AKR1C3 is expressed in normal tissues and plays a role in the carboxyl metabolism of a broad range of substrates, overexpression has been reported in many solid tumors including pancreatic cancer, gastric cancer, hepatocellular carcinoma (HCC), castrate-resistant prostate cancer, and adenocarcinoma and squamous cell carcinoma of the lung and gastroesophageal junction.^3–7^ There is evidence that AKR1C3 contributes to malignancy through its roles in cellular differentiation, migration, and proliferation.^8–11^ High expression levels of AKR1C3 in small cell lung cancer and HCC have been shown to correlate with poor survival prognosis.^9^^,^^12^ Increased AKR1C3 expression may also play a role in resistance to the chemotherapeutic agents abiraterone, doxorubicin, enzalutamide, and methotrexate, as well as in resistance to radiation therapy.^13–18^

**Figure 1.**
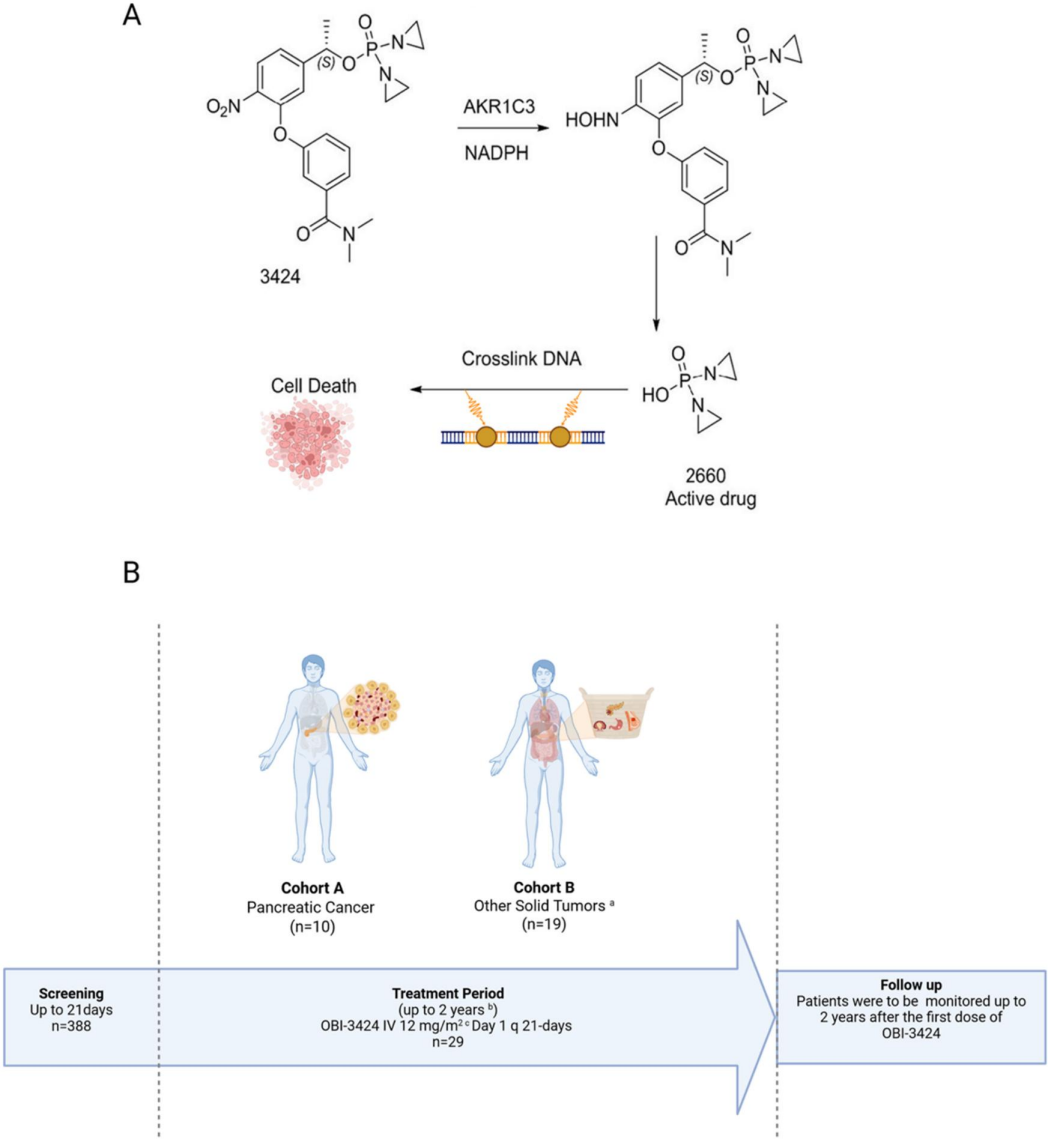
OBI-3424 mechanism of action and phase 2 trial design. (A) Figure has been modified from Tsimberidou et al.^22^; licensed under a Creative Commons Attribution 4.0 International License. http://creativecommons.org/licenses/by/4.0/. (B) Figure was created with BioRender.com. ^a^Any solid tumor other than pancreatic adenocarcinoma. ^b^Patients received treatment for up to 2 years (34 cycles) or until disease progression, unacceptable toxicity, or patient or physician decision, whichever occurred first.

OBI-3424, a small-molecule prodrug, is selectively activated by AKR1C3 in the presence of NADPH to become a potent DNA-alkylating agent (Figure 1A). Its intermediate form is spontaneously hydrolyzed to the aziridine bis-alkylating agent OBI-2660. OBI-2660 is cytotoxic, causing interstrand DNA crosslinking that results in cell death. Given the higher levels of AKR1C3 in cancers, OBI-3424 has the potential to be selectively activated in AKR1C3-expressing tumor cells while causing less damage to healthy tissues. Activation of OBI-3424 has been found to be highly specific for AKR1C3-expressing cells, distinguishing it and its potential safety profile from other prodrug alkylating agents such as cyclophosphamide and ifosfamide.^19^ OBI-3424-induced cytotoxicity correlates directly with cellular AKR1C3 expression levels in both cell-line and in vivo xenograft models.^19^ Cell-line and patient-derived xenograft mouse models demonstrate strong OBI-3424 anti-tumor activity at doses resulting in no observed toxicity in primates, suggesting that a safe and effective dose could be achievable in humans.^20^

**Figure 2.**
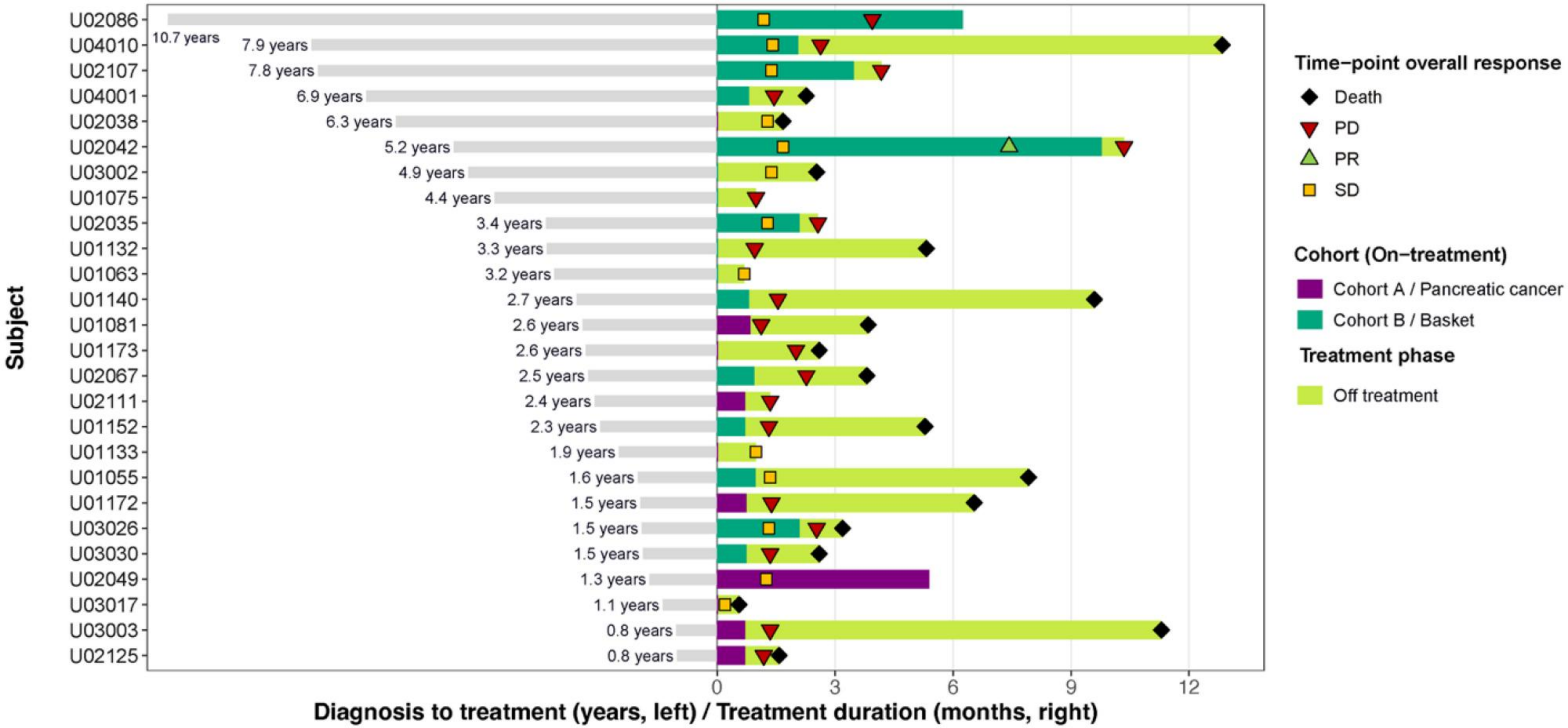
Swimmer plot showing disease course by patient (*N* = 26). PD, progressive disease; PR, partial response; SD, stable disease. The basket cohort included esophageal adenocarcinoma (U01055), rectosigmoid junction adenocarcinoma (U01075; U01152), urachal adenocarcinoma (U02042), malignant neoplasm of ampulla of Vater (U01132), and intrahepatic cholangiocarcinoma (U02086).

**Figure 3.**
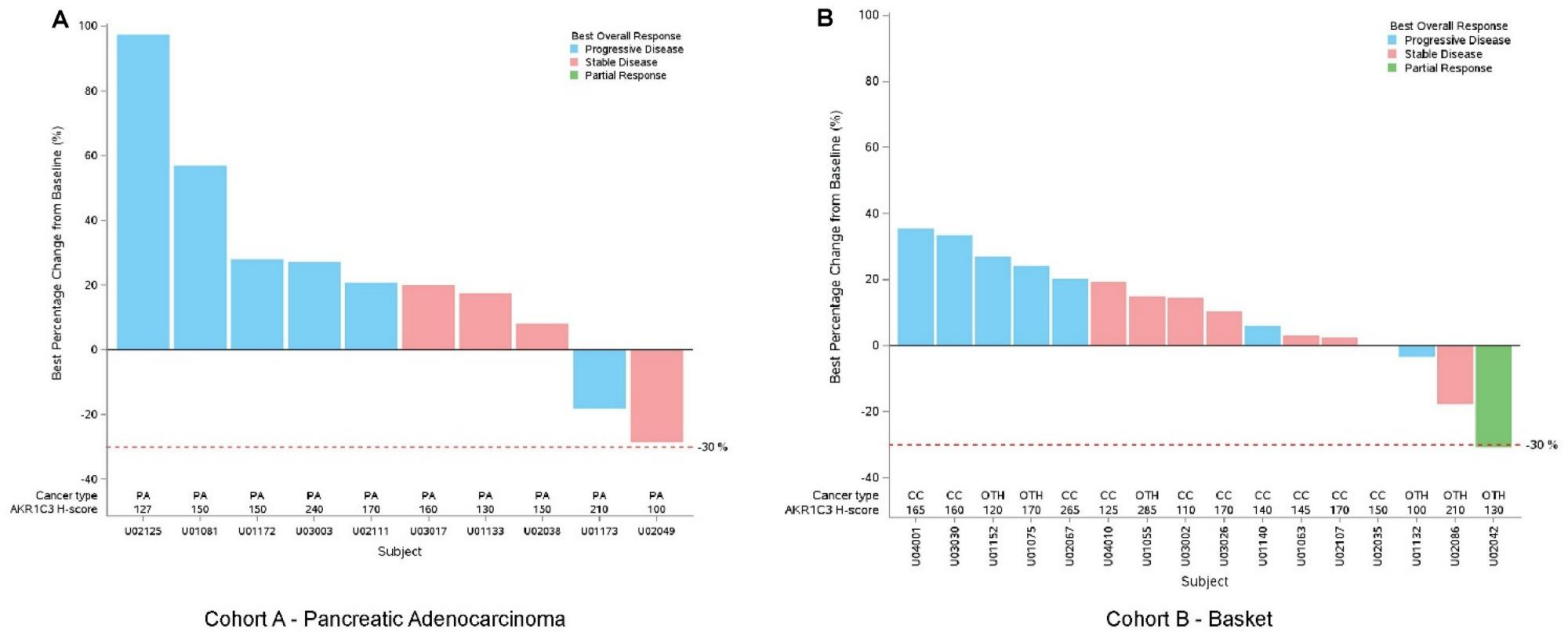
Best percentage change from baseline in target lesions (*n* = 26). AKR1C3, aldo-keto reductase family 1 member C3; CC, colorectal cancer; OTH, other solid tumors; PA, pancreatic adenocarcinoma. Other cancer types included esophageal adenocarcinoma (U01055), rectosigmoid junction adenocarcinoma (U01075; U01152), urachal adenocarcinoma (U02042), malignant neoplasm of ampulla of Vater (U01132), and intrahepatic cholangiocarcinoma (U02086).

**Figure 4.**
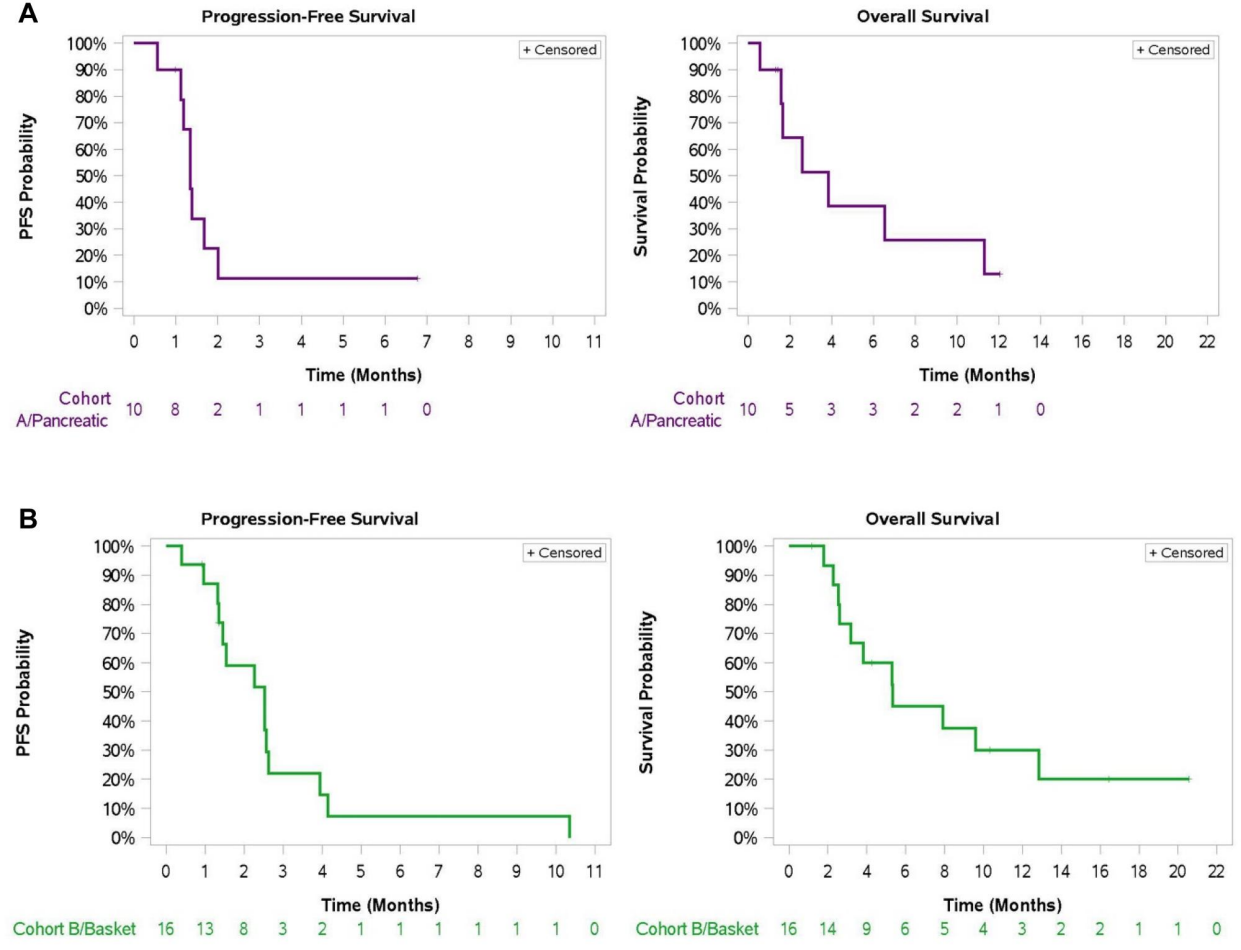
Kaplan-Meier curves for progression-free survival and overall survival. Progression-free survival (PFS) and overall survival (OS) curves by tumor cohort (pancreatic adenocarcinoma [panel A] and basket [panel B]) for patients treated with OBI-3424. Median PFS and OS are indicated for each cohort.

In 2018, OBI-3424 was granted an Orphan Drug Designation for the treatment of T-cell acute lymphoblastic leukemia and hepatocellular carcinoma (HCC) by the US Food and Drug Administration,^21^ and is being investigated in clinical trials. A phase 1/2 clinical trial of OBI-3424 is currently enrolling patients with relapsed/refractory T-cell acute lymphoblastic leukemia (NCT04315324) in collaboration with the Southwest Oncology Group. OBI-3424 is also being investigated in a phase 2 trial in patients with HCC.

The phase 1 dose-escalation portion of a phase 1/2 first-in-human, open-label, monotherapy trial of OBI-3424 (NCT03592264) evaluated its safety, tolerability, pharmacokinetics, and pharmacodynamics in patients with advanced solid tumors and provided evidence that a dose of 12 mg/m^2^ once every 21 days was well tolerated, although noncumulative thrombocytopenia and anemia were dose-limiting.^22^ Here we report the results of the phase 2 dose-expansion portion of the phase 1/2 OBI-3424 monotherapy trial in patients with advanced solid tumors.^22^ This dose-expansion study characterized the safety and efficacy of OBI-3424 in patients with measured moderate to high AKR1C3 expression with either pancreatic adenocarcinoma (pancreatic cancer cohort) or various other cancer types (basket cohort).

**Table:** 

**TRIAL INFORMATION**	
**Disease**	Advanced solid tumors (pancreatic adenocarcinoma cohort and basket cohort) with AKR1C3 IHC H-score ≥100
**Stage of disease/treatment**	Advanced/metastatic (predominantly Stage IV)
**Prior therapy**	Median 2 prior lines (pancreatic cohort; range 1-5); median 4 prior lines (basket cohort; range 1-12), standard of care
**Type of study**	Phase 2 dose-expansion (open-label, multi-center, monotherapy); Simon’s 2-stage design; NCT03592264
**Primary endpoints**	Objective response rate (ORR) per RECIST v1.1 in the pancreatic adenocarcinoma and basket cohorts
**Secondary endpoints**	Disease control rate (DCR), progression-free survival (PFS), overall survival (OS), and safety/tolerability (TEAEs per CTCAE v5.0)
**Additional details of endpoints or study design** Best response was assessed using the response evaluation criteria in solid tumors (RECIST) version 1.1. Patients were enrolled into 1 of 2 cohorts using a Simon’s 2-stage design: a pancreatic adenocarcinoma cohort and another solid tumor type (“basket”) cohort.	

**Table:** 

**DRUG INFORMATION**	
**Generic/working name**	OBI-3424 (also designated AST-3424)
**Company name**	OBI Pharma USA, Inc., San Diego, CA
**Drug type**	Small-molecule prodrug/DNA-alkylating agent
**Drug class**	AKR1C3-activated prodrug; aziridine bis-alkylating agent (active metabolite OBI-2660)
**Dose**	12 mg/m² (recommended Phase 2 dose from Phase 1 dose-escalation portion)
**Route**	IV
**Schedule of administration**	Day 1 of each 21-day cycle; up to 34 cycles or until PD, unacceptable toxicity, or patient/physician decision

**Table:** 

**DOSE-ESCALATION TABLE**			
**Tumor type**	**Dose of drug**	**Number enrolled**	**Number evaluable for toxicity**
**Pancreatic cancer**	12 mg/m^2^	10	10
**Any (basket)**	12 mg/m^2^	19	19

## Primary assessment method

**Table:** 

**Primary assessment method**	
**Title**	Objective response rate (ORR; CR + PR) and disease control rate (DCR; CR + PR + SD) per RECIST v1.1 in the evaluable population
**Number of patients screened**	388
**Number of patients enrolled**	29 (pancreatic adenocarcinoma cohort, *n* = 10; basket cohort, *n* = 19)
**Number of patients evaluable for toxicity**	29
**Number of patients evaluated for efficacy**	26 (evaluable population: patients with ≥1 on-treatment tumor assessment; pancreatic adenocarcinoma, *n* = 10; basket, *n* = 16)
**Evaluation method**	Tumor response by RECIST v1.1; PFS and OS by Kaplan-Meier method; AKR1C3 H-score vs. tumor change by Spearman’s correlation
Outcome notes Pancreatic cancer cohort: All 10 patients were treated and evaluable for response. Four patients (40.0%) had SD (Figures 2 and 3), and the remaining patients had PD. The disease control rate was 40.0% (95% CI, 12.2%-73.8%; Table S1). No association was observed between AKR1C3 H-score and change in tumor measurements (best response by RECIST, Spearman’s correlation *P* = .9597) (Figure 3). The median PFS duration was 1.35 months (95% CI, 0.56-2.00 months), and the median overall survival (OS) duration was 3.8 months (95% CI, 0.6-11.3 months) (Figure 4A). Basket cohort: Of the 19 patients treated, 16 were evaluable for response; patients lacking tumor response assessments were not considered evaluable. The ORR was 6.3%, and the disease control rate was 56.3% (95% CI, 29.9%-80.3%; Table S1). One patient (6.3%) with stage 4 urachal adenocarcinoma had a PR that lasted for 2.96 months (Figures 2 and 3). The patient was a 54-year-old White male who had metastases in the lung and lymph node, had received 1 line of prior chemotherapy (6 cycles total), and had an AKR1C3 H-score of 130. The patient’s treatment ended after 14 cycles due to PD at day 315. Eight patients (42.1%) had SD. The remaining patients had PD. No association was observed between the AKR1C3 H-score and change in tumor measurements (best response by RECIST, Spearman’s correlation *P* = .3644) (Figure 3). The median PFS duration was 2.53 months (95% CI, 1.35-2.63 months), and the median OS duration was 5.32 months (95% CI, 2.53-12.85 months) (Figure 4B).	

## Pharmacokinetics and pharmacodynamics

Pharmacokinetic and pharmacodynamic data from this dose-expansion phase were not reported separately; PK/PD characterization was conducted in the phase 1 dose-escalation portion of the trial.^22^ No new PK/PD analyses were performed in the current phase 2 expansion cohorts.

## Additional details of study design

### Patients

Eligible patients were aged ≥18 years and had advanced solid tumors and measurable disease per the Response Evaluation Criteria in Solid Tumors (RECIST) version 1.1. Patients were required to have an Eastern Cooperative Oncology Group (ECOG) performance status of 0 or 1 and tumors with moderate-to-high AKR1C3 expression, defined as an H-score ≥100 using a validated immunohistochemical assay (as previously described^22^). AKR1C3 testing was conducted centrally by NeoGenomics Lab (Aliso Viejo, CA USA), using an anti-AKR1C3 mouse monoclonal antibody (Sigma-Aldrich, clone NP6.G6.A6). Exclusion criteria included prior radiotherapy to more than 25% of the bone marrow, symptomatic brain metastases, previously treated other malignancies (except adequately treated nonmelanoma skin cancer, in situ cancer, or other cancers without the potential to interfere with the safety or efficacy assessment of the current trial), and HCC. Detailed eligibility criteria are available in the Supplementary Methods.

### Trial design

This was the phase 2 dose-expansion portion of an open-label, multicenter, phase 1/2 dose-escalation and dose-expansion trial of OBI-3424 monotherapy (Figure 1). This portion of the trial was conducted to obtain a preliminary assessment of efficacy and further characterize the safety of OBI-3424.^22^ The trial included a prescreening period, a screening period (up to 21 days), a treatment period, and a study termination visit (1-2 weeks after the last dose of OBI-3424). Patients were contacted for survival and subsequent cancer therapy information up to 2 years after the first dose of OBI-3424.

This dose-expansion portion of the trial was conducted at 5 centers in the United States. All patients provided written informed consent prior to any protocol-related activities. The trial was approved by independent ethics committees and/or institutional review boards before it was initiated and was conducted in accordance with the ethical principles of the International Council for Harmonization Guideline for Good Clinical Practice, the Declaration of Helsinki, and applicable local regulations. The trial was registered at www.clinicaltrials.gov (NCT03592264; July 19, 2018).

### Treatment

OBI-3424 was administered by intravenous (IV) infusion (through a central venous catheter, when feasible, with monitoring for extravasation) on Day 1 of each 21-day cycle at a dose of 12 mg/m^2^, which was the recommended phase 2 dose determined in the phase 1 portion of the trial.^22^ Patients received treatment for up to 2 years (34 cycles) or until progressive disease (PD), unacceptable toxicity, or a patient or physician decision to discontinue, whichever occurred first.

### Statistical analysis

Descriptive statistics were used for continuous and categorical variables and included medians and 95% confidence intervals (CIs). PFS and OS analyses were performed using the Kaplan-Meier method. The association between AKR1C3 H-score and change in tumor measurements was calculated using the Spearman correlation test (significance level *P* < .05).

The safety population was defined as all enrolled patients who received at least one dose of the study drug. Efficacy analyses were completed on the evaluable population, which comprised of all subjects who had at least one on-treatment tumor assessment. Adverse events (AEs) were defined as any undesirable event occurring during the study regardless of its relationship to the study drug and included time periods beginning after first administration of the study drug until 30 days after the last dose of the study drug. Serious adverse events (SAEs) were AEs that resulted in outcomes of death (unless death was due to disease progression), life-threatening adverse experiences, persistent or significant disability or incapacity, inpatient hospitalization, congenital anomaly or birth defects, or other medically important events that may require medical or surgical intervention to prevent one of the previously listed outcomes. Severity was determined in accordance with the grading scale presented in CTCAE version 5.0.^23^ Whether there was reasonable possibility that the study drug caused or contributed to the AE was assessed based on time between onset and drug administration, possible biologic mechanisms for the study drug causing or contributing to the AE, and whether there was another more likely cause of the AE including it being attributable to concurrent/underlying illness, other drugs, or procedures. The safety population was defined as enrolled patients who received at least 1 dose of the study drug. Efficacy analyses were completed on the evaluable population, comprised of all subjects who had at least 1 on-treatment tumor assessment.

## Results

### Patients

Overall, 388 patients were assessed for eligibility for the dose-expansion phase of the trial, and 29 (7.5%) patients were treated with OBI-3424 (10 patients in the pancreatic adenocarcinoma cohort and 19 patients in the basket cohort) (Figure S1, see online supplementary material for a color version of this figure). Among the 360 patients who did not meet the study criteria, the most common reasons for screening failure were low AKR1C3 expression (H-score <100) or inadequate tumor tissue for testing (*n* = 215, 55.4%), patient ineligibility for treatment (*n* = 60, 15.5%), and worsening performance status or death (*n* = 49, 12.6%).

### Pancreatic adenocarcinoma cohort (*n* = 10)

#### Baseline characteristics

The baseline characteristics of patients in the pancreatic adenocarcinoma cohort are presented in Table 1. The median age was 61.0 years (range, 44-67 years), and the median body mass index (BMI) was 21.4 kg/m^2^ (range, 17.0-40.3 kg/m^2^). There were 4 men and 6 women in this cohort, and the patients were predominantly White (7/10, 70.0%). Nine patients (90.0%) had an ECOG performance status (PS) of 1. Patients were heavily pretreated, with a median of 2 lines of prior therapy (range, 1-5). The median AKR1C3 H-score was 150 (range, 100-240).

**Table 1:** Patient characteristics.

	Pancreatic adenocarcinoma cohort (*N* = 10)	Basket cohort (*N* = 19)	Overall (*N* = 29)
**Age, median (range), years**	61.0 (44-67)	60.0 (37-84)	61.0 (37-84)
**Gender, *n* (%)**			
** Male**	4 (40.0)	13 (68.4)	17 (58.6)
** Female**	6 (60.0)	6 (31.6)	12 (41.4)
**Ethnicity, *n* (%)**			
** Not Hispanic or Latino**	10 (100)	19 (100)	29 (100)
**Race, *n* (%)**			
** Caucasian or White**	7 (70.0)	18 (94.7)	25 (86.2)
** African American or Black**	1 (10.0)	1 (5.3)	2 (6.9)
** Asian**	1 (10.0)	0	1 (3.4)
** Other**	1 (10.0)	0	1 (3.4)
**BMI^a^ median (range), kg/m^2^**	21.4 (17.0-40.3)	26.1 (19.6-39.0)	25.9 (17.0-40.3)
**ECOG performance status, *n* (%)**			
** 0**	1 (10.0)	2 (10.5)	3 (10.3)
** 1**	9 (90.0)	17 (89.5)	26 (89.7)
**AKR1C3 H-score, median (range)**	150.0 (100-240)	150.0 (100-285)	150.0 (100-285)
**Time from diagnosis to treatment, median (range), months**	18.0 (9.5-75.4)	40.0 (17.5-95.3)	30.6 (9.5-95.3)
**Prior lines of therapy, median (range)**	2 (1-5)	4 (1-12)	4 (1-12)
**Tumor types, *n* (%)**			
** Colorectal cancer**	NA	11 (57.9)	11 (37.9)
** Lung cancer**	NA	1 (5.3)	1 (3.4)
** Other^b^**	NA	7 (36.8)	7 (24.1)

Abbreviations: BMI, body mass index; ECOG, Eastern Cooperative Oncology Group; NA, not applicable.

a Data for body mass index were available for 25 patients (pancreatic adenocarcinoma cohort, *n* = 9; basket cohort, *n* = 16).

b Other tumors included esophageal adenocarcinoma, rectosigmoid junction adenocarcinoma, urachal adenocarcinoma, malignant neoplasm of ampulla of Vater, intrahepatic cholangiocarcinoma, and gastroesophageal junction adenocarcinoma.

#### Treatment

Patients received a median of 2 treatment cycles (range, 1-7) of OBI-3424, and the median cumulative dose was 36.2 mg (range, 16.5-95.4 mg). Time-on-treatment ranged from 0.1 to 23.4 weeks.

#### Safety

Seven patients (70.0%) experienced grade 3-4 treatment-emergent adverse events (TEAEs); no TEAE led to death. Eight (80.0%) patients experienced TEAEs that were considered possibly related to OBI-3424 (grade 2, 3/10 [30.0%]; grade 3, 3/10 [30.0%]; grade 4, 2/10 [20.0%]). The most common treatment-related TEAEs were anemia (4/10, 40.0%), fatigue (4/10, 40.0%), decreased platelet count (4/10, 40.0%), and nausea (4/10, 40.0%). Two patients (20.0%) experienced serious adverse events that were considered related to OBI-3424 but did not lead to study discontinuation. One of these 2 patients had received 4 lines of prior chemotherapy and had lung, liver, retroperitoneal space, superior mesenteric lymph node, and breast metastases. The patient experienced 2 episodes of grade 3 lower gastrointestinal hemorrhage that occurred 21 and 25 days after receiving a single dose of OBI-3424. The cause of these episodes was considered multifactorial: baseline medications, severe thrombocytopenia, and possibly OBI-3424. The episodes resolved, and the patient was taken off trial because of PD. The second patient had 2 lines of prior therapy and experienced grade 3 anemia and thrombocytopenia that were attributed to OBI-3424. The patient completed 7 cycles of treatment and ended treatment due to unacceptable toxicity or significant adverse events not related to the study drug.

### Basket cohort (*n* = 19)

#### Baseline characteristics

The median age of patients in the basket cohort was 60.0 years (range, 37-84 years), and the median BMI was 26.1 kg/m^2^ (range, 19.6-39.0 kg/m^2^) (Table 1). There were 13 men and 6 women, most of whom were White (18/19, 94.7%). Seventeen (89.5%) patients had an ECOG PS of 1. Patients were heavily pretreated, with a median of 4 lines of prior therapy (range, 1-12). The median AKR1C3 H-score was 150 (range, 100-285).

#### Treatment

Patients received a median of 2 treatment cycles (range, 1-14), and the median cumulative dose was 41.5 mg (range, 17.5-228.4 mg). Time-on-treatment ranged from 0.1 to 42.6 weeks.

#### Safety

Fifteen (78.9%) patients experienced grade 3-4 TEAEs, and no TEAE led to death. TEAEs considered at least possibly related to OBI-3424 occurred in 17 (89.5%) patients (grade 1, 1/19 [5.3%]; grade 2, 4/19 [21.1%]; grade 3, 10/19 [52.6%]; grade 4, 2/19 [10.5%]), the most common being anemia (8/19, 42.1%), fatigue (8/19, 42.1%), and thrombocytopenia (8/19, 42.1%). One patient with stage 4 colorectal carcinoma metastatic to the lungs experienced grade 3 dyspnea that occurred 54 days after initiation of treatment, which was considered related to the study drug. The event resolved and the patient remained in the trial on a reduced dose until disease progression.

## Discussion

This is the first report of the phase 2 trial of OBI-3424 in patients with advanced solid tumors. Overall, OBI-3424 demonstrated a favorable safety and tolerability profile. The disease control rate was 40.0% in the pancreatic adenocarcinoma cohort and 56.3% in the basket cohort. The best overall response was PR, observed in 1 patient (3.8%) with urachal adenocarcinoma. The most common TEAEs were cytopenia and fatigue (Table 2), which were likely due, in part, to the patients being heavily pretreated, having low bone marrow reserve, and the expression of AKR1C3 in bone marrow cells.^4^

**Table 2:** Serious adverse events (SAEs).

	Pancreatic adenocarcinoma cohort (*N* = 10) *n* (%)	Basket cohort (*N* = 19) *n* (%)	Overall (*N* = 29) *n* (%)
**TEAEs**	10 (100)	19 (100)	29 (100)
**Treatment-related TEAEs**	8 (80.0)	17 (89.5)	25 (86.2)
**Severe (grade 3-4) TEAEs**	7 (70.0)	15 (78.9)	22 (75.9)
**Serious TEAEs**	7 (70.0)	9 (47.4)	16 (55.2)
**TEAEs leading to study drug withdrawal**	1 (10.0)	2 (10.5)	3 (10.3)
**TEAEs leading to study discontinuation**	1 (10.0)	1 (5.3)	2 (6.9)
**TEAEs of infusion reactions**	0	0	0
**TEAEs leading to death**	0	0	0

Most common treatment-related TEAEs (≥10% of patients in any cohort)						
	Any grade	Grade ≥3	Any grade	Grade ≥3	Any grade	Grade ≥3
**Anemia**	4 (40.0)	3 (30)	8 (42.1)	7 (36.8)	12 (41.4)	10 (34.5)
**Fatigue**	4 (40.0)	0	8 (42.1)	2 (10.5)	12 (41.4)	2 (6.9)
**Thrombocytopenia**	4 (40.0)	3 (30)	8 (42.1)	5 (26.3)	12 (41.4)	8 (27.6)
**Nausea**	4 (40.0)	1 (10)	3 (15.8)	0	7 (24.1)	1 (3.4)
**Anorexia**	1 (10.0)	0	3 (15.8)	0	4 (13.8)	0
**Thrombocytopenia**	1 (10.0)	1 (10)	3 (15.8)	1 (5.3)	4 (13.8)	2 (6.9)
**Vomiting**	3 (30.0)	1 (10)	1 (5.3)	0	4 (13.8)	1 (3.4)
**Leucopenia**	3 (30.0)	2 (20)	1 (5.3)	0	4 (13.8)	2 (6.9)
**Constipation**	1 (10.0)	0	2 (10.5)	0	3 (10.3)	0
**Weight loss**	0	0	3 (15.8)	0	3 (10.3)	0

There were no objective responses in the pancreatic adenocarcinoma cohort, and the objective response rate in the basket cohort was 6.3%; these results are similar to those noted in the phase 1 dose-escalation portion of the trial (2.6%). The trial was terminated early due to a lack of clinical efficacy that led to changes in the sponsor’s development plans. The trial termination was not due to safety concerns.

The safety profile was similar to that reported in the phase 1 dose-escalation portion of the trial,^22^ and there were no safety concerns associated with the trial termination.

Various factors may have contributed to the lack of OBI-3424 efficacy observed in this trial. Patients were required to have moderate-to-high AKR1C3 H-scores for enrollment in the trial; however, no association was observed between AKR1C3 H-score and change in tumor measurements (best response by RECIST). The AKR1C3 H-score cutoff was determined based on the half-maximal inhibitory concentration (IC_50_) of OBI-3424 observed in vitro. This cutoff limited enrollment and may have excluded patients who might have responded to OBI-3424 treatment, as responses were seen in patients with lower H-scores in the dose-expansion portion of the trial.^22^

Another explanation for the lack of efficacy is that the drug may not have efficiently reach AKR1C3-rich targets to induce tumor cell death. The tumor microenvironment is complex, with a dense extracellular matrix, interstitial pressure, and vessel compression, all of which can significantly impede drug delivery through the tumor vasculature,^24^^,^^25^ particularly in pancreatic cancer, which is resistant to therapeutics in part due to its dense tumor microenvironment.^26^ Immunosuppressive conditions can also be a barrier to drug delivery, resulting in limited therapeutic efficacy.^27^^,^^28^ OBI-3424 may also be more effective in combination with other therapies in addressing inherent tumor heterogeneity.^23^^,^^29^ This trial was conducted in a small number of patients, a limitation that may impact the generalizability of the trial results. As enrollment occurred during the global COVID-19 pandemic, delays in the testing of tumor samples and other pandemic-related issues negatively impacted the number of patients transitioning from initial screening to treatment.

OBI-3424 demonstrated favorable safety and tolerability, but its limited clinical efficacy in this phase 2 dose-expansion trial contributed to the early termination of the trial. These results highlight the challenges in developing novel targeted anticancer therapeutics. Ongoing trials are expected to provide additional data regarding the clinical efficacy of OBI-3424 in T-cell acute lymphoblastic leukemia and HCC.

## Supplementary Material

oyag254_Supplementary_Data

## Data Availability

The data supporting the findings of this study are available within the main text and Supplementary Material. All requests for further data sharing will be reviewed by MD Anderson and the study sponsors to determine whether the request is subject to any intellectual property or confidentiality obligations. For further questions, please contact the corresponding author at atsimber@mdanderson.org.
