# Supplementary material for: Phase 2 dose-expansion trial of OBI-3424, a DNA-alkylating prodrug, in patients with advanced solid tumors expressing AKR1C3
Source: Oncologist. 2026 Jul 2;31(8):oyag254. doi: 10.1093/oncolo/oyag254 (PMC13372677; doi:10.1093/oncolo/oyag254)
Supplement: oyag254_Supplementary_Data [file oyag254_supplementary_data.docx]

**Supplemental Methods**

**Eligibility Criteria**

Patients must have met all of the following criteria to be included in the study (both initial dose escalation phase and current expansion phase, unless otherwise specified):

Inclusion Criteria

1. At least 18 years of age
2. Ability to understand the purposes and risks of the study and has signed a written informed consent form approved by the investigator's Institutional Review Board (IRB)/Independent Ethics Committee (IEC)
3. Recovered from toxicities of prior therapy to Grade 0 or 1
4. Measurable disease by Response Evaluation Criteria in Solid Tumors (RECIST version 1.1) criteria
5. Eastern Cooperative Oncology Group (ECOG) performance status of 0 or 1
6. Acceptable liver function:
   1. Bilirubin ≤1.5 × institutional ULN
   2. AST and ALT ≤3.0 × ULN, or ≤5.0 × ULN for subjects with liver involvement
7. Acceptable renal function:
   1. Creatinine clearance >30 mL/min according to the Cockcroft-Gault formula
8. Acceptable hematologic status (without hematologic support, other than red blood cell transfusion)
   1. ANC ≥1500 cells/μL
   2. Platelet count ≥100,000/μL
   3. Hemoglobin ≥9.0 g/dL (prior packed red blood cell transfusion or erythropoietin support is allowed)
9. Females of childbearing potential must not have had unprotected sexual intercourse within 30 days before study entry and must agree to use a highly effective method of contraception (i.e., total abstinence, an intrauterine device, a double-barrier method [such as condom plus diaphragm with spermicide], a contraceptive implant, or an oral contraceptive) or have a vasectomized partner with confirmed azoospermia throughout the entire study period and for 30 days after study drug discontinuation.

Inclusion Criteria for Expansion Phase Subjects Only:

1. Available tumor tissue, either archival or fresh (fresh preferred).
2. For treatment, an AKR1C3 IHC H-score of ≥ 100 using a validated IHC assay in one of the following tumor types to be enrolled in the respective cohort:
   1. Cohort A: Pancreatic adenocarcinoma
   2. Cohort B: Basket (any solid tumor type other than pancreatic adenocarcinoma)

Exclusion Criteria

1. Prior radiotherapy to more than 25% of the bone marrow
2. Symptomatic brain metastases, unless previously treated and well controlled for at least 4 weeks after central nervous system (CNS)-directed treatment as ascertained by clinical examination and brain imaging (magnetic resonance imaging [MRI] or computed tomography [CT]) during the Screening Period. Patients with known leptomeningeal disease are excluded.
3. Previously treated malignancies, except for adequately treated non-melanoma skin cancer, in situ cancer, or other cancers whose natural history or treatment does not have the potential to interfere with the safety or efficacy assessment of the current study
4. Patients with hepatocellular carcinoma (applies to Expansion Phase only)
5. Major surgery, other than diagnostic surgery, within 4 weeks prior to Day 1, without complete recovery
6. Active, uncontrolled bacterial, viral, or fungal infections requiring systemic therapy
7. Treatment with radiation therapy, surgery, chemotherapy, targeted therapies, or hormones within 3 weeks prior to study entry (6 weeks for nitrosoureas or mitomycin C)
8. Concomitant use of strong CYP3A4 inhibitors/inducers
9. Concomitant use of naproxen within a 48-hour window before and after OBI-3424 dosing
10. Females who are pregnant or breast-feeding
11. Concomitant disease or condition that could interfere with the conduct of the study, or that would, in the opinion of the investigator, pose an unacceptable risk to the subject in this study
12. Unwillingness or inability to comply with the study protocol for any reason.

**Supplemental Table 1. Antitumor activity in patients treated with OBI-3424 (evaluable population)**

|  | **Pancreatic Adenocarcinoma Cohort (N=10)** | **Basket Cohort  (N=16)** | **Overall (N=26)** |
| --- | --- | --- | --- |
| **Best overall tumor response,^a^ n (%)** |  |  |  |
| Partial response | 0 | 1 (6.3) | 1 (3.9)^b^ |
| Stable disease | 4 (40.0) | 8 (50.0) | 12 (46.2) |
| Progressive disease | 6 (60.0) | 7 (43.8) | 13 (50.0) |
| **Objective response rate**  n (%)  95% CI | 0  (0, 30.8) | 1 (6.3)  (0.2, 30.2) | 1 (3.9)  (0.1, 19.6) |
| **Disease control rate ^c^** |  |  |  |
| n (%) | 4 (40.0) | 9 (56.3) | 13 (50.0) |
| 95% CI | (12.2, 73.8) | (29.9, 80.3) | (29.9, 70.1) |

^a^ Patients without tumor response assessments were treated as not evaluable for best overall response.

^b^ Patient was diagnosed with urachal adenocarcinoma.

^c^ Disease control rate was defined as complete response + partial response + stable disease.

CI, confidence interval.

**Supplemental Figure 1. Patient Flow Diagram**

**Expansion**

Considered for enrollment (underwent tumor prescreening for AKR1C3)

N=388

Enrolled subjects

N=29

Received OBI-3424^a^

N=29

- Cohort A (pancreatic), n=10
- Cohort B (basket), n=19

Failed screening

N=360

- Low AKR1C3 expression or inadequate tumor for testing, n=215
- Ineligible for treatment, n=60
- Subject withdrawal, n=20
- Unknown, n=16
- Worsening status/death, n=49

^a^ One patient failed screening but received treatment.
